# NPHS Mutations in Pediatric Patients with Congenital and Steroid-Resistant Nephrotic Syndrome

**DOI:** 10.3390/ijms252212275

**Published:** 2024-11-15

**Authors:** Jun Xin Lee, Yan Jin Tan, Noor Akmal Shareela Ismail

**Affiliations:** 1 Department of Pediatrics, Faculty of Medicine, Universiti Kebangsaan Malaysia, Cheras, Kuala Lumpur 56000, Malaysia; junxin@ukm.edu.my; 2 Department of Biochemistry, Faculty of Medicine, Universiti Kebangsaan Malaysia, Cheras, Kuala Lumpur 56000, Malaysia; tanyanjin97@gmail.com

**Keywords:** NPHS1, NPHS2, congenital nephrotic syndrome, steroid-resistant nephrotic syndrome, pediatric, end-stage renal failure

## Abstract

NPHS1 and NPHS2 are kidney gene components that encode for nephrin and podocin, respectively. They play a role in the progression of congenital (CNS) and steroid-resistant (SRNS) nephrotic syndrome. Hence, this study aimed to determine the prevalence and renal outcomes of NPHS mutations among pediatric patients with CNS and SRNS. We also aimed to identify potential predictors of NPHS mutations in this patient cohort. Overall, this study included 33 studies involving 2123 patients screened for NPHS1, whereas 2889 patients from 40 studies were screened for NPHS2 mutations. The patients’ mean age was 4.9 ± 1 years (ranging from birth to 18 years), and 56% of patients were male (n = 1281). Using the random-effects model, the pooled proportion of NPHS1 mutations among pediatric patients with CNS and SRNS was 0.15 (95% CI 0.09; 0.24, *p* < 0.001, I^2^ = 92.0%). The pooled proportion of NPHS2 mutations was slightly lower, at 0.11 (95% CI 0.08; 0.14, *p* < 0.001, I^2^ = 73.8%). Among the 18 studies that reported ESRF, the pooled proportion was 0.47 (95% CI 0.34; 0.61, *p* < 0.001, I^2^ = 75.4%). Our study showed that the NPHS1 (β = 1.16, *p* = 0.35) and NPHS2 (β = 5.49, *p* = 0.08) mutations did not predict ESRF in CNS and SRNS pediatric patients. Nevertheless, patients from the European continent who had the NPHS2 mutation had a significantly higher risk of developing ESRF (*p* < 0.05, β = 1.3, OR = 7.97, 95% CI 0.30; 2.30) compared to those who had the NPHS1 mutation. We recommend NPHS mutation screening for earlier diagnosis and to avoid unnecessary steroid treatments. More data are needed to better understand the impact of NPHS mutations among pediatric patients with CNS and SRNS.

## 1. Introduction

Nephrotic syndrome (NS) is a form of kidney disease among children who have impaired glomerular function. It manifests clinically through massive proteinuria, hypoalbuminemia, edema, and dyslipidemia [1,2]. Such patients respond well to corticosteroids in most cases. This leads to fairly good outcomes. However, about 15–20% of these patients are steroid-resistant, referred to as steroid-resistant NS (SRNS) [3,4]. This is a serious problem because it often causes progressive worsening in renal function, with up to 50% of children developing end-stage renal failure (ESRF) within 15 years of diagnosis [5,6].

Congenital nephrotic syndrome (CNS) is an extreme form of this condition that occurs either in the uterus or within 3 months of birth [7,8]. It can be very severe, and it can be diagnosed early. CNS has worse outcomes than SRNS as the majority of these patients die from ESRF by the time they are between two to three years old, showing high morbidity rates [8,9]. Treatment for CNS usually involves aggressive measures, such as albumin infusions, nephrectomies, and, eventually, renal transplantation, which is still the gold-standard treatment modality for preventing death and improving the quality of life and life expectancy in affected patients [9,10,11].

With the progression of genetic research, our understanding of the molecular basis of CNS and SRNS has greatly expanded. Over fifty genes have been implicated in these disorders so far, with many of them encoding proteins that are essential in constructing and maintaining the glomerular filtration barrier (GFB), especially the slit diaphragm [12,13,14]. The latter is a specialized cell–cell junction within the glomerulus, composed of several key proteins, including nephrin and podocin, which are encoded by NPHS1 and NPHS2, respectively [15].

Nephrin is a structural component of the slit diaphragm that acts as a filter protein, while podocin is one of its close interacting partners [16,17]. Nephrin serves as a major structural protein in the formation of the filtration-selective slit structure, and it makes up its backbone [17]. Podocin is another important constituent molecule. It interacts closely with nephrin and other structural components to help maintain this important structure. Mutations on either NPHS1 or NPHS2 lead to the malfunctioning of the slit diaphragm, causing podocyte collapse, alteration in glomerular permeability, and, hence, nephrotic syndrome’s main feature such as proteinuria. ESRF progression occurs rapidly among the patients harboring these mutations due to the severe and progressive disruption of glomerular function.

Autosomal recessive inheritance is seen in both NPHS1 and NPHS2 mutations; thus, two copies of the mutated gene (one from each parent) are necessary for the disease to manifest [18]. CNS was first identified within Finnish populations, hence the name “Finnish type nephrotic syndrome”, which is commonly associated with NPHS1 mutations [19]. However, NPHS1 mutations are found not only in Finland but also in other countries across the globe.

On the contrary, it has been reported that Caucasian and Asian individuals are predominantly affected by NPHS2 mutations [20]. Nevertheless, mutation analyses conducted among pediatric patients affected by SRNS originating from different ethnic backgrounds show significant variations in the prevalence rate of NPHS2 mutations, varying between 10% and 60% [21]. Furthermore, some populations, like children with CNS, have almost equal frequencies of the occurrence of both NPHS1 and NPHS2 mutations [22]. Some studies, however, reveal an absence of virtually any NPHS1 mutation among populations such as Turkish children [23], indicating the complexity and heterogeneity of these genetic disorders.

The importance of genotype–phenotype correlations in pediatric patients with CNS and SRNS is emphasized by the variation in NPHS1 and NPHS2 mutations that occur among different populations. Despite these mutations being identified, the clinical predictors of NPHS mutations are poorly understood, and specific genotypes have not been well-connected to clinical outcomes. This knowledge gap is a major challenge in managing and predicting CNS and SRNS, as well as in developing targeted therapies.

Against this background, we aim to undertake this systematic review and meta-analysis to determine the prevalence and renal outcomes associated with the NPHS1 and NPHS2 mutations in pediatric patients with CNS and SRNS. By evaluating all available data, this research will attempt to provide further insight into understanding the genetic basis of these diseases, identify possible indicators for mutation occurrence, or the absence thereof, and establish definitive relations between genotypes and phenotypes. The possible applications of these results may involve genetic screening programs, early diagnosis improvement initiatives, and treatment customization, meant to improve prognosis in children who suffer from these severe kidney diseases.

## 2. Results

### 2.1. Systematic Search Results

**Figure 1:**
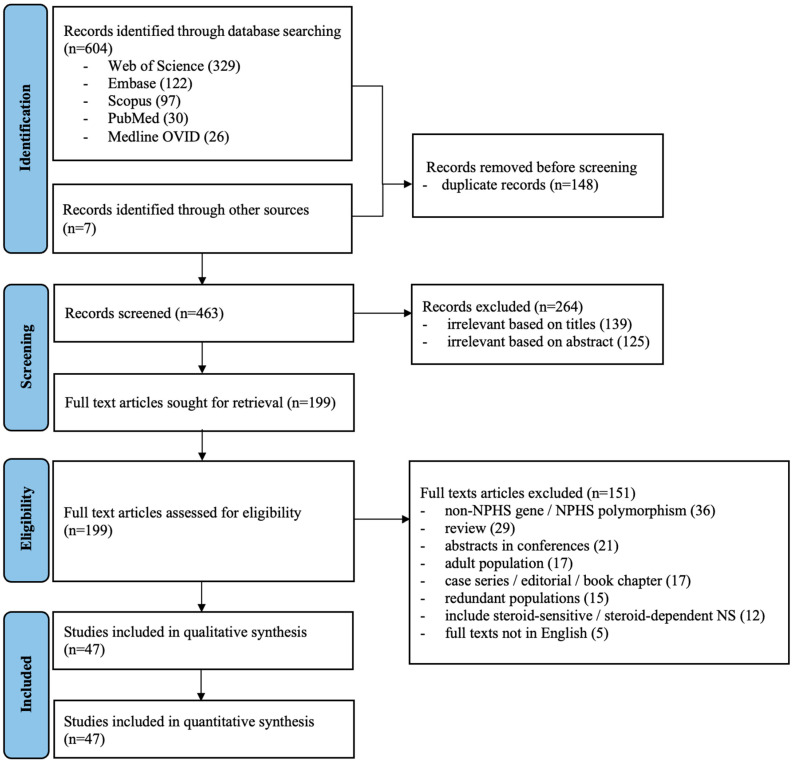
Flow chart of the study selection process (flow chart adapted from PRISMA).

The searches from electronic databases yielded 604 articles while a manual search through the references of the included articles found an additional 7 articles (Figure 1); 463 titles and abstracts were screened after removing 148 duplicates. Overall, 199 full-text articles were retrieved and reviewed for eligibility. Eventually, 47 studies were included which consisted of 13 cohorts, 4 case–controls, and 30 cross-sectional studies. The methodological quality of all the studies was summarized in Supplementary Table S1.

Overall, there were 33 studies with 2123 patients screened for NPHS1 (Table 1), whereas 2889 patients from 40 studies were screened for NPHS2 mutations (Table 2). The patients’ mean age was 4.9 ± 1 years (ranging from birth to 18 years) with 56% male patients (n = 1281); 50% of patients were from the Asian continent.

### 2.2. Prevalence of NPHS1 and NPHS2 Mutations in CNS and SRNS Pediatric Patients

**Figure 2:**
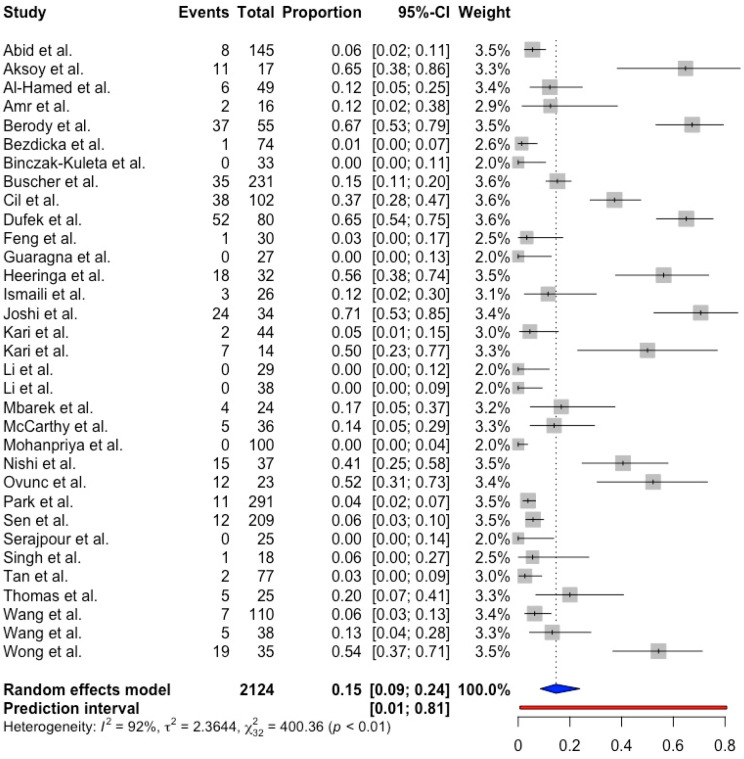
Forest plot showing NPHS1 mutation in CNS and SRNS pediatric patients [8,9,18,21,24,25,26,27,28,29,30,31,32,33,34,35,36,37,38,39,40,41,42,43,44,45,46,47,48,49,50,51,52].

**Figure 3:**
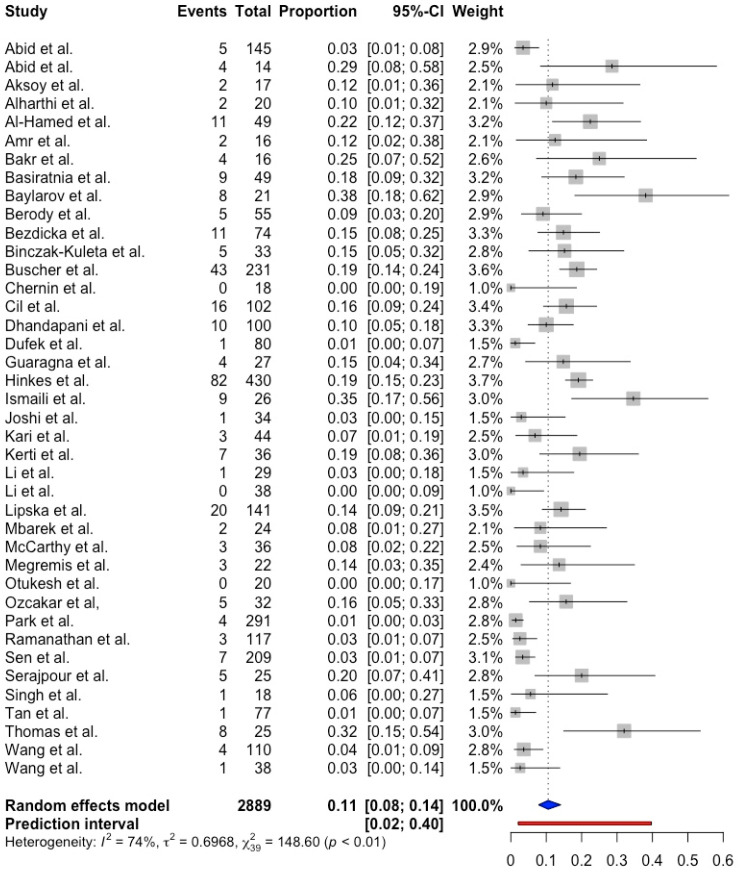
Forest plot showing NPHS2 mutation in CNS and SRNS pediatric patients [8,9,18,20,21,24,25,26,27,28,29,31,33,34,35,37,38,39,40,44,45,46,47,48,49,50,51,53,54,55,56,57,58,59,60,61,62,63,64,65].

Using the random effects model, the pooled proportion of NPHS1 mutations among pediatric patients with CNS and SRNS was 0.15 (95% CI 0.09; 0.24, prediction interval 0.01; 0.81, *p* < 0.001) with substantial between-study heterogeneity (I^2^ = 92.0%). (Figure 2). The pooled proportion of NPHS2 mutations was 0.11 (95% CI 0.08; 0.14, prediction interval 0.02; 0.40, *p* < 0.001) with substantial between-study heterogeneity (I^2^ = 73.8%) (Figure 3).

### 2.3. ESRF in CNS and SRNS Pediatric Patients with NPHS Mutations

**Figure 4:**
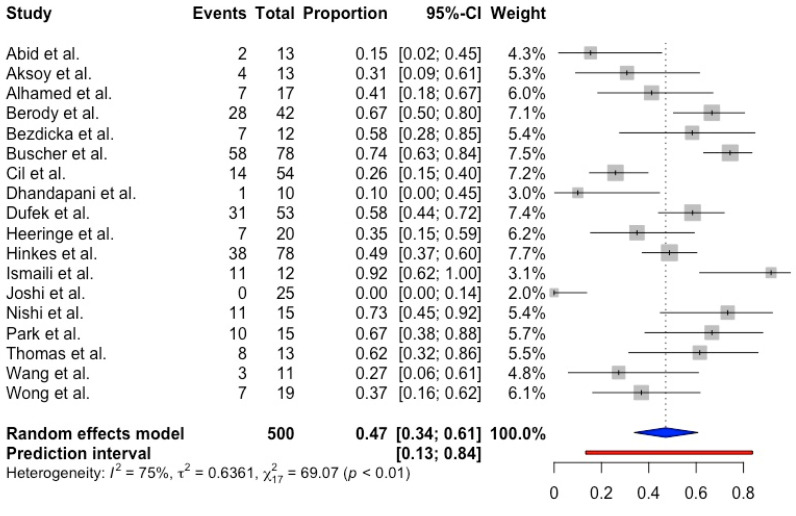
Forest plot showing ESRF in CNS and SRNS pediatric patients with NPHS mutations [8,9,21,24,25,26,28,29,32,33,34,42,44,49,50,51,58,59].

**Figure 5:**
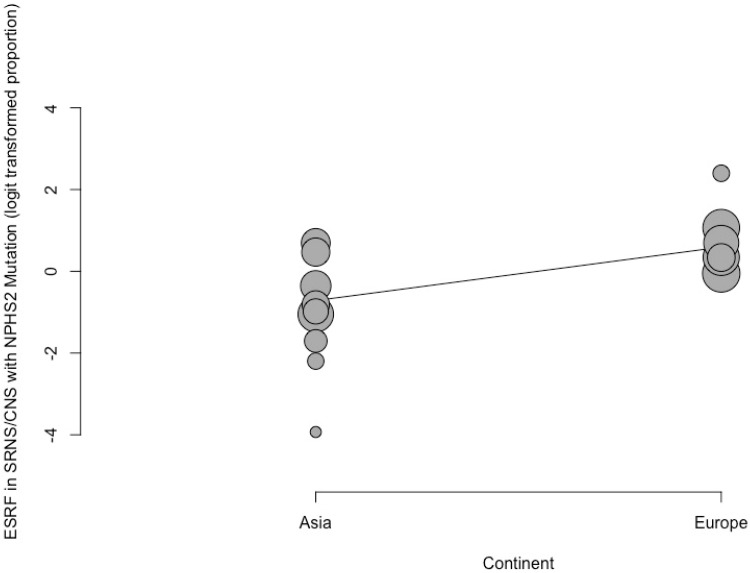
Meta-regression bubble plot of ESRF in CNS and SRNS pediatric patients with NPHS mutations against country continents.

In total, 18 studies reported on ESRF in pediatric patients with NPHS mutations. The pooled proportion of ESRF in this cohort was 0.47 (95% CI 0.34; 0.61, prediction interval 0.13; 0.84, *p* < 0.001) with substantial between-study heterogeneity (I^2^ = 75.4%) (Figure 4). Our meta-regression showed that both the NPHS1 (regression coefficient = 1.16, *p* = 0.35) and NPHS2 (regression coefficient = 5.49, *p* = 0.08) mutations did not predict ESRF in pediatric patients with CNS and SRNS. Further subgroup analysis and meta-regression revealed that children with the NPHS2 mutation from the European continent had a significantly higher risk of developing ESRF (*p* < 0.05, β = 1.3, OR = 7.97, 95% CI 0.30; 2.30), but not children with the NPHS1 mutation (Figure 5).

## 3. Discussion

Nephrotic syndrome is a significant cause of chronic kidney disease (CKD) in the pediatric population, with a worldwide incidence of approximately 4.7 per 100,000 individuals [66,67]. Clinically, it is characterized by the triad of heavy proteinuria, hypoalbuminemia, and peripheral edema. Most pediatric patients diagnosed with idiopathic nephrotic syndrome respond favorably to corticosteroid therapy, leading to a relatively good prognosis. However, a subset of these patients, approximately 15% to 20%, do not respond to steroids, resulting in what is known as steroid-resistant nephrotic syndrome (SRNS) [68,69]. The incidence of SRNS is about 3–4 per million person-years, which represents a critical clinical challenge due to its association with poor long-term renal outcomes [3].

The understanding of the pathophysiology of SRNS and CNS has significantly evolved with the discovery of various causative genes that affect different structural and functional components of the kidney, particularly the glomerular filtration barrier. This barrier is composed of the glomerular basement membrane, slit diaphragm, and podocytes, each playing a crucial role in maintaining the kidney’s filtering capacity.

**Figure 6:**
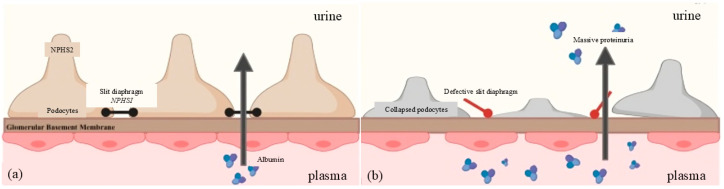
(**a**) Role of NPHS1 and NPHS2 in glomerular filtration in normal kidney and (**b**) impact of NPHS1 and NPHS2 mutations in SRNS/CNS pediatric patients.

NPHS1 and NPHS2 are major genes involved in SRNS and CNS (Figure 6). Located on chromosome 19 (19q13.1), NPHS1 possesses 29 exons [70]. It codes for nephrin, a vital cell adhesion molecule that belongs to the immunoglobulin superfamily (Figure 6a). Nephrin functions critically in maintaining the structural integrity of the slit diaphragm, which is a specific structure within the glomerulus responsible for ultrafiltration, thereby preventing protein loss into urine such as albuminuria [17,71]. Any faults in NPHS1 disrupt this important role, hence leading to proteinuria, which characterizes nephrotic syndrome.

Podocin, a protein expressed mainly in podocytes, is encoded by NPHS2 located on chromosome 1 (1q25-q31) [72]. It plays a major role in maintaining the integrity of the slit diaphragm. By interacting with nephrin and other proteins, it ensures the normal functioning of the slit diaphragm [17]. Abnormalities in both the slit diaphragm and podocytes occur due to NPHS2 mutations, which lead to severe proteinuria and, most often, a fast progression to ESRF (Figure 6b).

The finding of mutations in NPHS1 and NPHS2 has important implications for the clinical management of SRNS and CNS. The PodoNet Registry, a comprehensive web-based database spanning 28 countries mainly across Europe, has brought about a significant understanding about these disorders at molecular levels [3]. The EURenOmics project was carried out among over 500 steroid-resistant patients with documented genetic testing using next-generation sequencing (NGS) and Sanger sequencing, both aimed at a systematic screening of known genes causing podocytopathy [73]. Over thirty specific genes for podocytes have been reported until now; however, mutations in NPHS1 as well as NPHS2 are more common among SRNS/CNS patients.

Neonatal proteinuria is caused by genetic mutations in either NPHS1 or NPHS2. In SRNS and CNS patients, the PodoNet Registry reports 12.6% NPHS1 mutations [3], while our systematic review reported NPHS1 mutation rates in Asian populations which were about 15%. Conversely, the prevalence of NPHS2 mutations in our review was 11%, which is significantly lower than the 41.8% registered by the PodoNet database [3]. This difference may be ascribed to a higher frequency of NPHS2 mutations seen among European-derived populations where the knowledge and availability of new advanced genetic technologies are more common.

These genetic mutations have far-reaching clinical implications. The PodoNet cohort has demonstrated that the overall ESRF-free survival among SRNS patients decreases with time: 74% at 5 years, 58% at 10 years, and 48% at 15 years [3]. A genetic diagnosis increases the risk of progressing to ESRF by about 150% [3,74]. Interestingly, long-term renal outcomes appear similar between children with different genetic mutations such that there appears to be no difference in ten-year survival rates between NPHS2-associated nephropathy and other WT1-associated nephropathy genotypes [3]. This is in concordance with our results, which showed that NPHS mutations do not predict ESRF. However, patients from the European continent with NPHS2 mutation had an eight-times significantly higher risk of progressing to ESRF. This could be due to the higher prevalence of the NPHS2 mutation in European countries.

The modality of genetic testing encompasses the traditional Sanger sequencing, followed by the rapid advancement of next-generation sequencing (NGS) [75]. NGS approaches for genetic diagnosis include the gene panel, whole-exome sequencing, and whole-genome sequencing [75]. Sanger sequencing has been the gold standard, providing high sensitivity and specificity, especially for small-size genes. Identifying the genotype–phenotype correlation data as reported in our systematic review could aid in providing a genetic diagnosis. Gene-panel analysis is used as an indication-driven mutation analysis by including genes which are associated with certain phenotypes; hence, it is a more cost-effective approach [76]. Whole-exome and whole-genome sequencing provide a more thorough genetic analysis of the exome and genome, respectively, and, thus, require a higher cost. The high cost has been the major challenge in utilizing genetic testing for the genetic diagnosis of SRNS/CNS.

However, the discovery and identification of the NPHS1 and NPHS2 mutations have several implications on individual health as well as family planning. To patients diagnosed with NPHS, this knowledge will help them make sound clinical decisions in avoiding steroid treatment which is unnecessary and bears long-term side effects. Early genetic diagnosis permits personalized treatments that may include alternative therapies tailored to a given genetic mutation.

From the standpoint of family planning, identifying examples of NPHS mutations provides vital information for susceptible families. Genetic testing through prenatal diagnosis enables early interventions like genetic counseling and consideration for reproductive options such as preimplantation genetic diagnosis (PGD). In this way, they can be used in subsequent generations to prevent the transmission of severe nephrotic syndromes, thereby improving long-term outcomes for affected families.

In addition, an appreciation of where these mutations exist geographically underscores the need for increasing awareness and access to genetic testing in regions with a lower reported prevalence like Asia. Increasing the availability of advanced genetic testing technologies including NGS could bring about earlier and more accurate diagnoses, ultimately improving outcomes for children with SRNS and CNS globally.

## 4. Materials and Methods

### 4.1. Study Protocol and Guideline

This systematic review and meta-analyses were conducted in concordance with the Preferred Reporting Items for Systematic Reviews and Meta-Analyses (PRISMA) guidelines [77]. The study protocol was registered in the International Prospective Register of Systematic Reviews (PROSPERO) (CRD42022346280) (accessed on 24 July 2022).

### 4.2. Search Strategy and Study Selection Criteria

The following databases were used to perform systematic searches: SCOPUS, PubMed, OVID (MEDLINE), Embase, and Web of Science. The combinations of search terms were as follows: #1: “genes” [MeSH] AND “Nephrotic syndrome, idiopathic, steroid-resistant” [Supplementary Concept] AND “Pediatrics” [MeSH]; and #2: “NPHS1” [Free Text Term] OR “NPHS2” [Free Text Term] AND “Nephrotic syndrome, idiopathic, steroid-resistant” [Supplementary Concept] AND “Pediatrics” [MeSH]. Manual searching for relevant references from published articles was also conducted. The last search was conducted on the 31 May 2023.

Two independent reviewers identified the studies extracted from database searches. All citation records were managed with EndNote(R) version 20 (The EndNote Team, Philadelphia, PA, USA) and duplications were removed. Relevant articles were screened independently by the authors through research titles, abstracts, and index terms of the manuscripts. Articles that reported sufficient information on NPHS1 and NPHS2 mutations in pediatric patients with CNS and SRNS with the following study designs were included: systematic reviews, cohort studies, case–control studies, and analytical cross-sectional studies. Case series, case reports, editorials or commentaries, and in-vitro studies were excluded. Pediatric patients without NPHS1 and NPHS2 mutations or CNS and SRNS were also excluded. The most recent publication was included for published articles that reported results from overlapping populations.

### 4.3. Data Collection and Methodological Quality Assessment

Full-text articles that fulfilled the inclusion criteria were retrieved to assess their eligibility. Included citations were exported to an Excel spreadsheet and coded information as follows: author/s, study title, study design, country of study, year of publication, DOI, sample size, gender, ethnicity, age of diagnosis, NPHS1 and NPHS2 mutations, renal biopsy, types of mutation, ESRF, and mortality. Data extracted from each article were cross-checked by two independent investigators, whilst an independent third reviewer provided the final decision in the event of any disagreement.

The methodological quality of all included articles was assessed using the Joanna Briggs Institute (JBI) critical appraisal tools for the respective study designs [78], and further classified into poor (0–49%), moderate (50–69%), or high (70% and above) quality [79]. This was carried out independently by two authors (LJX, TYJ) and a third author (NASI) resolved any dispute.

### 4.4. Operational Definitions

In this review, NPHS1 and NPHS2 mutations referred to homozygous and compound heterozygous mutations that were pathogenic or likely pathogenic based on the American College of Medical Genetics (ACMG) genetic variants classification [80]. Single heterozygous mutations and polymorphisms were not included because of the autosomal recessive nature of NPHS1 and NPHS2 mutations. ESRF was defined as a glomerular filtration rate (GFR) of less than 15 mL/min/1.73 m^2^ following the Kidney Disease Improving Global Outcomes (KDIGO) classification [81]. The pediatric population in this review was up to 18 years old.

### 4.5. Statistical Analysis

R packages including metafor version 3.0, meta, and dmetar were used for meta-analysis and meta-regression [82,83]. Both packages were implemented in R version 4.2.1 (R Core Team, Vienna, Austria) using RStudio desktop version 2022.07.0+548 (RStudio Team, Boston, MA, USA).

Pooled proportions with logit transformations were computed using the random effects models for the following: (i) NPHS1 and NPHS2 mutations in pediatric patients with CNS and SRNS and (ii) ESRF in CNS and SRNS patients with NPHS1 and NPHS2 mutations. Restricted Maximum Likelihood (REML) and the Hartung–Knapp adjustments were used to estimate the variances, τ^2^ for both effect measures, and to calculate the confidence intervals of summary effects, respectively. The 95% confidence interval for τ^2^ was obtained using the Q-profile method.

The Cochran’s Q test was used to assess the heterogeneity and *p* < 0.1 indicates significant heterogeneity, whilst *l*^2^ statistic was classified as follows: (i) 0–40%: possibly unimportant; (ii) 30–60%: moderate heterogeneity; (iii) 50–90%: substantial heterogeneity; and (iv) 75–100%: considerable heterogeneity [84]. Subgroup and sensitivity analyses were performed in the presence of substantial heterogeneity (Cochran χ^2^ *p* < 0.1 or *l*^2^ > 50%).

Publication bias was evaluated by subjective visual inspection of the funnel plots and objectively by Egger’s test. The Galbraith plot was used to detect the presence of outlier studies.

NPHS1 and NPHS2 mutations were evaluated as predictors for ESRF in pediatric patients with CNF and SRNS using meta-regression. All tests were two-tailed, and *p*-values of 0.05 or lower were statistically significant.

## 5. Conclusions

NPHS mutations do not predict ESRF in pediatric patients with SRNS and CNS as our study indicates. Despite that, European pediatric patients with the NPHS2 mutation had an eight-times significantly higher risk of progressing to ESRF. A high between-study effect in terms of heterogeneity for the NPHS mutation results was also observed, although our sensitivity analysis showed no “outlying” studies which may lead to this. Hence, we postulate that this high heterogeneity could be attributed to other factors such as the method of genetic testing, the time frame of genetic analysis, and differences in the study population. More data are needed to find out other dependable predictors of NPHS mutations and ESRF within this patient group.

In summary, the understanding of SRNS and CNS has completely changed since the discovery of NPHS1 and NPHS2 mutations, which have provided insights into their pathophysiology and provided new opportunities for personalized treatment and family planning. There is hope that these findings will help improve the lives of children around the world who suffer from these complex diseases as genetic research continues to evolve.

## Figures and Tables

**Table 1:** Characteristics of studies included for NPHS1 mutation.

No.	Author (Year)	Study Type	Country	N	NPHS1 Mutation, n (%)	Age in Years, Median (Range)	Male, n (%)	ESRD, n (%)	Mortality, n (%)
1.	Abid et al. (2012) [21]	Cross-sectional	Pakistan	145	8 (5.5)	(0–1)	4 (50.0)	1 (12.5)	1 (5.0)
2.	Aksoy et al. (2019) [24]	Retrospective cohort	Turkey	17	11 (64.7)	0 (0–0.25)	7 (63.6)	4 (36.4)	0 (0)
3.	Al-Hamed et al. (2013) [25]	Cross-sectional	Saudi Arabia	49	6 (12.2)	0.2 (0.2–2)	4 (66.7)	2 (33.3)	0 (0)
4.	Amr et al. (2020) [18]	Case–control	Egypt	16	2 (12.5)	0.17 (0.17)	1 (50.0)	NR	NR
5.	Berody et al. (2018) [9]	Prospective cohort	France	55	37 (67.3)	0 (0–1.1)	20 (54.1)	25 (67.6)	6 (16.2)
6.	Bezdicka et al. (2018) [26]	Prospective cohort	Czech Republic, Slovakia	74	1 (1.4)	0.1	0	0	0
7.	Binczak-Kuleta et al. (2014) [27]	Retrospective cohort	Poland	33	0 (0)	-	-	-	-
8.	Buscher et al. (2016) [28]	Prospective cohort	Germany	231	35 (15.2)	NR	19 (54.3)	29 (82.9)	0 (0)
9.	Cil et al. (2014) [29]	Prospective cohort	Multinational	102	38 (37.3)	1.1 ± 0.1	16 (42.1)	8 (21.0)	14/29 (48.3)
10.	Dufek et al. (2018) [8]	Retrospective cohort	Multinational	80	52 (65)	0.9 (0.4–3.8)	26 (50.0)	31 (59.6)	7 (13.5)
11.	Feng et al. (2014) [30]	Case–control	China	30	1 (3.3)	4.8	0 (0)	0 (0)	0 (0)
12.	Guaragna et al. (2015) [31]	Cross-sectional	Brazil	27	0 (0)	-	-	-	-
13.	Heeringa et al. (2008) [32]	Cross-sectional	Multinational	32	18 (56.3)	0 (0–0.2)	9 (50)	7 (35)	1 (5)
14.	Ismaili et al. (2008) [33]	Retrospective cohort	Belgium	26	3 (11.5)	0	NR	3 (100)	0 (0)
15.	Joshi et al. (2021) [34]	Cross-sectional	India	34	24 (70.6)	0 (0–0.3)	12 (50)	0 (0)	0 (0)
16.	Kari et al. (2013) [35]	Retrospective cohort	Saudi Arabia	44	2 (4.5)	1.8 (1.5–2.0)	0 (0)	NR	NR
17.	Kari et al. (2014) [36]	Retrospective cohort	United Kingdom	14	7 (50)	0.1 (0–0.3)	NR	7 (100)	0 (0)
18.	Li et al. (2019) [37]	Cross-sectional	China	29	0 (0)	-	-	-	-
19.	Li et al. (2019) [38]	Retrospective cohort	China	38	0 (0)	-	-	-	-
20.	Mbarek et al. (2010) [39]	Cross-sectional	Tunisia	24	4 (16.7)	0.2 (0–0.3)	4 (100)	4 (100)	4 (100)
21.	McCarthy et al. (2013) [40]	Cross-sectional	United Kingdom	36	5 (13.9)	0.2 (0–11)	2 (40.0)	1 (20)	0 (0)
22.	Mohanapriya et al. (2018) [41]	Case–control	India	100	0 (0)	-	-	-	-
23.	Nishi et al. (2019) [42]	Retrospective cohort	Japan	36	15 (41.7)	0 (0–0.1)	5 (33.3)	11 (73.3)	0 (0)
24.	Ovunc et al. (2017) [43]	Cross-sectional	Multinational	23	12 (52.2)	0.1 (0–0.2)	6 (50)	NR	NR
25.	Park et al. (2020) [44]	Cross-sectional	Korea	291	11 (3.8)	0 (0)	7 (63.6)	7 (63.6)	2 (18.2)
26.	Sen et al. (2017) [45]	Cross-sectional	Multinational	209	12 (5.7)	0 (0–2)	7 (58.3)	NR	NR
27.	Serajpour et al. (2019) [46]	Cross-sectional	Iran	25	0 (0)	-	-	-	-
28.	Singh et al. (2021) [47]	Cross-sectional	India	18	1 (5.6)	1.3	1 (100)	0 (0)	0 (0)
29.	Tan et al. (2018) [48]	Cross-sectional	United States	77	2 (2.6)	0.2 (0.1–0.3)	0	2 (100)	0 (0)
30.	Thomas et al. (2022) [49]	Cross-sectional	Egypt	25	5 (20.0)	0.4 (0.3–0.6)	3 (60.0)	1 (20)	0 (0)
31.	Wang et al. (2017) [50]	Cross-sectional	China	110	7 (6.4)	1.5 (0–0.4)	4 (57.1)	0 (0)	2 (28.6)
32.	Wang et al. (2017) [51]	Cross-sectional	China	38	5 (13.2)	3 (1–7)	3 (60.0)	NR	NR
33.	Wong et al. (2013) [52]	Retrospective cohort	New Zealand	35	19 (54.3)	0 (0–0.7)	12 (63.2)	7 (36.8)	3 (15.8)

**Table 2:** Characteristics of studies included for NPHS2 mutations.

No.	Author (Year)	Study Type	Country	N	NPHS2 Mutation, n (%)	Age in Years, Median (Range)	Male, n (%)	ESRD, n (%)	Mortality, n (%)
1.	Abid et al. (2012) [21]	Cross-sectional	Pakistan	145	5 (3.4)	NR	4 (80)	1 (20)	0 (0)
2.	Abid et al. (2018) [53]	Cross-sectional	Pakistan	14	4 (28.6)	2 (2–3)	3 (75)	1 (25)	0 (0)
3.	Aksoy et al. (2019) [24]	Retrospective cohort	Turkey	17	2 (11.8)	0 (0)	0 (0)	0 (0)	0 (0)
4.	Alharthi et al. (2016) [54]	Cross-sectional	Saudi Arabia	20	2 (10)	7 (5–9)	0 (0)	2 (100)	0 (0)
5.	Al-Hamed et al. (2013) [25]	Cross-sectional	Saudi Arabia	49	11 (22.4)	2 (0.1–6)	5 (45.5)	5 (45.5)	0 (0)
6.	Amr et al. (2020) [18]	Case–control	Egypt	16	2 (12.5)	NR	NR	NR	NR
7.	Bakr et al. (2008) [55]	Cross-sectional	Egypt	16	4 (25.0)	NR	NR	0 (0)	0 (0)
8.	Basiratnia et al. (2013) [56]	Case–control	Iran	49	9 (18.4)	NR	NR	1 (6.7)	0 (0)
9.	Baylarov et al. (2020) [57]	Cross-sectional	Azerbaijan	21	8 (38.1)	NR	NR	0 (0)	0 (0)
10.	Berody et al. (2018) [9]	Prospective cohort	France	55	5 (9.1)	0 (0–0.6)	4 (80)	3 (60)	1 (20)
11.	Bezdicka et al. (2018) [26]	Prospective cohort	Czech Republic, Slovakia	74	11 (14.9)	0.4 (0–2.5)	6 (54.5)	7 (63.6)	0 (0)
12.	Binczak-Kuleta et al. (2014) [27]	Retrospective cohort	Poland	33	5 (15.2)	0.4 (0.1–8.3)	2 (40)	5 (100.0)	0 (0)
13.	Buscher et al. (2016) [28]	Prospective cohort	Germany	231	43 (18.6)	NR	20 (46.5)	29 (67.4)	0 (0)
14.	Chernin et al. (2008) [20]	Cross-sectional	United States	18	0 (0)	-	-	-	-
15.	Cil et al. (2014) [29]	Prospective cohort	Multinational	102	16 (15.6)	2.6 ± 0.8	10 (62.5)	6 (37.5)	0 (0)
16.	Dhandapani et al. (2016) [58]	Cross-sectional	India	100	10 (10.0)	NR	NR	1 (8.3)	1 (8.3)
17.	Dufek et al. (2018) [8]	Retrospective cohort	Multinational	80	1 (1.3)	0.7	1 (100)	0 (0)	0 (0)
18.	Guaragna et al. (2015) [31]	Cross-sectional	Brazil	27	4 (14.8)	7.1 (1.2–13)	1 (25.0)	1 (25.0)	0 (0)
19.	Hinkes et al. (2008) [59]	Cross-sectional	Multinational	430	82 (19.1)	1.6 (0–16.6)	45/78 (57.7)	38/78 (48.7)	NR
20.	Ismaili et al. (2008) [33]	Retrospective cohort	Belgium	26	9 (34.6)	5 (0–15)	NR	8 (88.9)	0 (0)
21.	Joshi et al. (2021) [34]	Cross-sectional	India	34	1 (2.9)	0	1 (100)	0 (0)	0 (0)
22.	Kari et al. (2013) [35]	Retrospective cohort	Saudi Arabia	44	3 (6.8)	3 (1.5–11)	NR	NR	NR
23.	Kerti et al. (2012) [60]	Cross-sectional	Hungary	36	7 (19.4)	4 (0.3–14)	3 (42.9)	4 (57.1)	0 (0)
24.	Li et al. (2019) [37]	Cross-sectional	China	29	1 (3.4)	2.6	1 (50)	0 (0)	0 (0)
25.	Li et al. (2019) [38]	Retrospective cohort	China	38	0 (0)	-	-	-	-
26.	Lipska et al. (2013) [61]	Cross-sectional	Poland	141	20 (14.2)	NR	NR	NR	NR
27.	Mbarek et al. (2010) [39]	Cross-sectional	Tunisia	24	2 (8.3)	12.5 (10–15)	0 (0)	2 (100)	1 (50)
28.	McCarthy et al. (2013) [40]	Cross-sectional	United Kingdom	36	3 (8.3)	2 (0.9–6)	1 (33.3)	0 (0)	0 (0)
29.	Megremis et al. (2009) [62]	Cross-sectional	Greece	22	3 (13.6)	4 (2–4)	1 (33.3)	2 (66.7)	0 (0)
30.	Otukesh et al. (2009) [63]	Cross-sectional	Iran	20	0 (0.0)	-	-	-	-
31.	Ozcakar et al. (2006) [64]	Cross-sectional	Turkey	32	5 (15.6)	5.5 (0.2–12)	3 (60)	NR	NR
32.	Park et al. (2020) [44]	Cross-sectional	Korea	291	4 (1.4)	1.7 (0–6.9)	3 (75)	3 (75)	0 (0)
33.	Ramanathan et al. (2016) [65]	Cross-sectional	India	117	3 (2.6)	3.0 (1.5–4.0)	2 (66.7)	0 (0)	0 (0)
34.	Sen et al. (2017) [45]	Cross-sectional	Multinational	209	7 (3)	11 (0.1–13)	4 (57)	NR	NR
35.	Serajpour et al. (2019) [46]	Cross-sectional	Iran	25	5 (20)	NR	NR	NR	NR
36.	Singh et al. (2021) [47]	Cross-sectional	India	18	1 (5.6)	0.6	1 (100)	0 (0)	0 (0)
37.	Tan et al. (2018) [48]	Cross-sectional	United States	77	1 (1.3)	3.2	0 (0)	1 (100)	0 (0)
38.	Thomas et al. (2022) [49]	Cross-sectional	Egypt	25	8 (32.0)	2.5 (1.2–1.8)	4 (50.0)	7 (87.5)	0 (0)
39.	Wang et al. (2017) [50]	Cross-sectional	China	110	4 (3.6)	2.6 (2.0–3.4)	2 (50)	3 (75)	0 (0)
40.	Wang et al. (2017) [51]	Cross-sectional	China	38	1 (2.6)	3	1 (100)	NR	NR

## Data Availability

The data of this article is available upon reasonable request from the corresponding author.

## Supplementary Materials

The following supporting information can be downloaded at: https://www.mdpi.com/article/10.3390/ijms252212275/s1.
