# Supplementary material for: NPHS Mutations in Pediatric Patients with Congenital and Steroid-Resistant Nephrotic Syndrome
Source: Int J Mol Sci. 2024 Nov 15;25(22):12275. doi: 10.3390/ijms252212275 (PMC11594456; doi:10.3390/ijms252212275)
Supplement: Supplementary file 1 [file ijms-25-12275-s001.zip › ijms-3258345-supplementary.pdf]

Supplementary Material

Supplementary Table S1. Methodological quality of included studies based on the JBI critical appraisal tools for each study design.

Author	Study Design	Item 1	Item 2	Item 3	Item 4	Item 5	Item 6	Item 7	Item 8	Item 9	Item 10	Item 11	Quality
Abid 2012	Cross-sectional	√	√	√	√	X	X	√	√				Good
Abid 2018	Cross-sectional	√	√	√	√	X	X	√	√				Good
Aksoy	Cohort	√	√	√	X	X	√	√	√	↔	X		Mod
Alharti	Cohort	√	√	√	↔	X	√	√	√	↔	X		Mod
Al-Hamed	Cross-sectional	√	√	√	√	X	X	√	√				Good
Amr	Case control	√	√	√	√	√	X	X	√	↔	√		Good
Bakr	Cross-sectional	√	√	√	√	X	X	√	√				Good
Basiratnia	Case control	√	√	√	√	√	X	X	√	↔	√		Good
Baylarov	Cross-sectional	√	√	√	√	X	X	√	√				Good
Berody	Cohort	√	√	√	↔	↔	√	√	√	↔	X	√	Mod
Bezdicka	Cohort	√	√	√	↔	X	√	√	√	√	√	√	Good
Binczak-Kuleta	Cohort	√	√	√	↔	X	√	√	√	↔	X	√	Mod
Buscher	Cohort	√	√	√	↔	X	√	√	√	↔	X	√	Mod
Chernin	Cross-sectional	√	√	√	√	X	X	√	√				Good
Cil	Cohort	√	√	√	↔	↔	√	√	√	↔	X	√	Mod
Dhandapani	Cross-sectional	√	√	√	√	X	X	√	√				Good
Dufek	Cohort	√	√	√	↔	↔	√	√	√	↔	X	√	Mod
Feng	Cross-sectional	√	↔	√	√	X	X	√	√				Mod
Guaragna	Cross-	√	↔	√	√	X	X	√	√				Mod

	sectional												
Hinkes	Cross-sectional	√	↔	√	√	X	X	√	√				Mod
Heeringa	Cross-sectional	√	↔	√	√	X	X	√	√				Mod
Ismaili	Cohort	√	√	√	X	X	√	√	√	√	X	√	Good
Joshi	Cross-sectional	√	√	√	√	X	X	√	√				Good
Kari 2013	Cohort	√	√	√	X	X	√	√	√	√	X	√	Good
Kari 2014	Cohort	√	√	√	X	X	√	√	√	√	X	√	Mod
Kerti	Cross-sectional	√	↔	√	√	X	X	√	√				Mod
Li	Cross-sectional	√	√	√	√	↔	X	√	√				Mod
Li	Cohort	√	√	√	X	X	√	√	√	√	X	√	Good
Lipska	Cross-sectional	√	↔	√	√	X	X	√	√				Mod
Mbarek	Cross-sectional	√	√	√	√	X	X	√	√				Good
McCarthy	Cross-sectional	√	↔	√	√	X	X	√	√				Mod
Megremis	Cross-sectional	√	↔	√	√	X	X	√	√				Mod
Mohanapriya	Case control	√	√	√	√	√	X	X	√	X	√		Good
Nishi	Cohort	√	√	√	↔	X	√	√	√	↔	X	√	Mod
Otukesh	Cross-sectional	√	√	√	√	X	X	√	√				Good
Ovunc	Cross-sectional	√	↔	√	√	X	X	√	√				Mod
Ozcakar	Cross-sectional	√	√	√	√	X	X	√	√				Good
Park	Cross-sectional	√	√	√	√	X	X	√	√				Good
Ramanathan	Cross-sectional	√	↔	√	√	X	X	√	√				Mod
Sen	Cross-	√	↔	√	√	X	X	√	√				Mod

	sectional												
Serajpour	Cross-sectional	√	↔	√	√	X	X	√	↔				Mod
Singh	Cross-sectional	√	↔	√	√	X	X	√	↔				Mod
Tan	Cross-sectional	√	√	√	√	X	X	√	√				Good
Thomas	Cross-sectional	√	√	√	√	↔	X	√	√				Good
Wang	Cross-sectional	√	√	√	√	X	X	√	√				Good
Wang	Cross-sectional	√	√	√	√	X	X	√	√				Good
Wong	Cohort	√	√	√	X	X	√	√	√	↔	X	√	Mod

√, *Yes; X, No;* ↔, *Unclear; NA, not applicable*
Good: 70% and above
Moderate: 50-69%
Poor: 0-49%
